# The health of single mothers and fathers in Germany

**DOI:** 10.17886/RKI-GBE-2017-123

**Published:** 2017-12-13

**Authors:** Petra Rattay, Elena von der Lippe, Lea-Sophie Borgmann, Thomas Lampert

**Affiliations:** Robert Koch Institute, Department of Epidemiology and Health Monitoring, Berlin

**Keywords:** SINGLE PARENTS, SINGLE-PARENT FAMILIES, PARTNERSHIP, HEALTH, HEALTH-RELATED BEHAVIOUR

## Abstract

In every fifth family in Germany, one parent lives alone with children in the household. Life as a single parent is often marked by challenges that include adopting sole responsibility for the child’s education and care, alongside employment commitments, and the difficulties of reconciling work and family life. Moreover, despite comparatively high employment rates, single parents – and their children – are greatly affected by poverty.

This paper compares the health of single parents and parents living in partnership and analyses the extent to which single parents’ health varies according to their socio-economic and employment status, and social support.

The analysis was conducted using data from the German Health Update (GEDA) study in 2009, 2010 and 2012 on fair or poor self-rated general health, as well as depression, back pain, obesity, smoking, sporting inactivity and the non-utilization of dental check-ups. The analyses are based on data from 9,806 women and 6,279 men living in a household with at least one child under the age of 18.

The study identified a significantly higher prevalence for all health indicators (apart from obesity) among single mothers compared to mothers living with a partner. In the case of single fathers, higher prevalences were found for depression, smoking and the non-utilization of dental check-ups. On average, the lower socio-economic status of women can explain a certain proportion of the health impairment of single parents, but not for men. However, a lower socio-economic status or social support do not account for the health impairments of single parents. Therefore, the higher prevalence of health impairments among single parents cannot simply be attributed to differences in employment status or to lower levels of social support; rather, certain health indicators show a cumulative effect between single-parents status and the social factors mentioned above.

The results presented here provide a differentiated view of the relationship between the health and social situation of single parents. Improving the financial position of one-parent families and making it easier to reconcile work and family life are important steps that would help improve the health of single parents.

## 1. Introduction

Although single parent families are, historically speaking, not a new occurrence, the social conditions for single parenting have changed considerably. The marked increase in single-parent families in Germany over the past few decades is an expression of the pluralisation of family forms [1]. Single parenthood has now become one of multiple accepted family types, albeit a temporary one that can change, for example, when a single parent begins a relationship with a new partner or when the child leaves the family home. In addition to a certain freedom of choice, single parents face the challenges that arise from having sole responsibility for managing the household, as well as bringing up children and, in many cases, employment commitments, limited financial and temporal resources, which can be accompanied by psychosocial problems and other health impairments.

According to the German microcensus, 1.46 million single mothers and 180,000 single fathers were living with one or more minor children in 2014 [2, 3]. Thus 20.3% of all families comprised single parents with children [4]. In 2014, 2.3 million children were growing up in a single-parent family [4]. In nine out of ten cases, the parent was the child’s mother. In addition, 53% of single mothers and 63% of single fathers were divorced or separated from their former partner; just 4% of single mothers were widows, and one in ten single fathers were widowers [2]. Single mothers are more likely to care for two or more children and younger children than single fathers: around one third of single mothers and just 12% of single fathers lived with children under the age of 6 [2, 5]. In 2014, 58% of single mothers, 58% of married mothers and 57% of unmarried mothers living with a partner were in employment. However, about four in ten single or cohabiting mothers in employment were full-time employees. In contrast, only about a quarter of married mothers were in full-time employment. Opposing trends were identified in terms of employment among fathers living with children under the age of 15: single fathers were significantly less likely to be in employment (70%) compared to married fathers (85%) and cohabiting fathers (80%). Similarly, whereas 86% of single fathers work full-time, this is the case for 95% of married fathers and 92% of cohabiting fathers [2].

According to the microcensus, the income poverty risk – defined as an income of less than 60% of the population’s median income (the needs-weighted median income) – was 42% among single-parent families in 2014 [4]. In 2015, 38% of single-parent families received benefits in accordance with the German Social Code (SGB II); this is about five times the rate found among two-parent families. Moreover, two thirds of single parents with three or more children received these benefits. 35% of all single parents who received benefits under SGB II in 2014 did not earn enough to support their families despite being in employment. Finally, about half of the 1.92 million children who received SGB II benefits lived in one-parent households [4].

Since the 1980s, a strand of research that is particularly established in Great Britain and the US has developed a focus on the health of single parents. This topic only gained attention in the area of research and politics in Germany in the early 2000s [6]. In Germany, published studies tend to concentrate on single mothers [6-14]. This is because of the larger proportion of single mothers and the very small number of single fathers represented in population-based surveys. However, a number of German studies, particularly on mental health, include results about both single mothers and fathers [15-18].

Considerable disparity exists in the way in which these studies define single parenthood (in terms of the age of the child/children living in a household), the social group with which single parents are compared (married mothers or mothers living with partners), the age of the participants in the sample, and the methodology (e.g. with regard to the included control variables). Despite this, all of the studies produced similar findings.

The studies show that single mothers rate their health as poor more frequently than mothers living with a partner [6, 8-10, 12]. In addition, when single mothers are affected by depression or disorders related to the use of psychoactive substances, such as alcohol and drugs, they face a higher burden than mothers in the same situation who are living with a partner [6-8, 10, 11, 15-17, 19, 20]. Single mothers also have a worse basic emotional state [6, 8, 9] and a lower health-related quality of life [9, 10].

Any differences that do exist between the physical health of single mothers and those living with a partner are usually only minor. For example, there are no significant differences in terms of hypertension [6, 10], dizziness [10], migraine [6, 9], chronic bronchitis [10], allergies [9], bronchial asthma [6], diseases of the uterus, ovaries or fallopian tubes [9], diabetes mellitus [6], cancer [6] or stroke [6]. Nevertheless, there are some indications that single mothers are more frequently affected by pain in their daily activities than mothers living with a partner [9, 10]. With regard to chronic back pain, Hancioglu [6] found significant differences between single mothers and those living with a partner; although the results published by Lange and Saß [10] and Hoffmann and Swart [8] do not corroborate these findings.

Regarding the health-related behaviour, research in Germany has focused on smoking and physical activity in particular. All of the studies identified a markedly higher prevalence of smoking among single mothers [9, 10, 12, 21, 22]. The studies also suggest that single mothers take part in sporting activity less often that mothers living with a partner [9, 12]. Despite this, there are only slight differences in terms of average body mass index (BMI) [8]. Only minor differences, if any, were identified for the utilization of medical services. However, psychotherapists are more frequently consulted by single mothers than by mothers living with a partner [8]. On the other hand, single mothers tend to attend medical check-ups less often [8, 9] and take psychotropic medicines, especially sleeping pills, more frequently than mothers living with a partner [9, 10].

In contrast, very little data is available on the health of single fathers in Germany. According to the Robert Koch Institute’s report on the health of men in Germany, single fathers are more likely to rate their general health as poor, more frequently report that they have been medically diagnosed with depression, and are more likely to smoke than fathers living with a partner [23]. Studies on mental health also show that single fathers face a heavy psychological burden [17, 18] and an even higher prevalence of mental disorders than single mothers [15, 16]. No differences were identified between single fathers and fathers living with a partner with regard to obesity and physical stress [18, 23].

A number of reasons have been put forward to explain the somewhat poorer levels of health among single parents. Single parents are solely responsible for the care and upbringing of their children. They also often face a precarious economic situation combined with the double burden of having family responsibilities alongside employment commitments. In addition, single parents also have fewer social and temporal resources at their disposal. Therefore, studies usually report health outcomes that are statistically controlled for various social parameters. Doing so it can be analysed whether single parenting is associated with health impairments, or whether these impairments can be attributed to the often less favourable social, financial and temporal resources. In summary, population-based studies in Germany that control for social and economic factors often identify a reduction in the differences in health between single parents and parents living in partnership [10, 12, 17, 18, 22], but the differences cannot be fully explained by the social situation.

Analyses that differentiate between subgroups of single mothers have identified significant differences in health depending on the level of social support and their financial situation [8, 9, 11]. Furthermore, a higher degree of satisfaction together with received social support [8, 19] or financial situation [9, 19], can have a ‘buffering’ effect on the health of single mothers. According to these studies, the stronger the social and financial resources of single mothers are, the better their health situation is. Differentiated analyses on the situation of single fathers are not available.

This paper provides an up-to-date description of selected parameters on the health of single mothers and fathers using data from a large population-based health survey. Due to the large sample size, the data from the Robert Koch Institute enables analyses on the health of single mothers and fathers. Based on previous research, indicators with high public health relevance for people aged between 18 and 59 were selected. In addition to general health (defined as fair, poor or very poor), the indicators include depression, back pain, obesity, smoking, sporting inactivity and the non-utilization of dental check-ups.

This paper particularly focuses on the following questions:

► How healthy are single parents and what differences are there in terms of health-related behaviour between single parents and parents living with a partner?
► Can differences in socio-economic status, employment status and social support explain the greater health impairment facing single parents?
► Is the association between single parenthood and health/health-related behaviour different for mothers and fathers?
► Are there differences between single mothers and mothers living with a partner in terms of the association between socio-economic status, employment status and social support, on the one hand, and health and/or health-related behaviour, on the other?

## 2. Methods

### 2.1 Data

The analyses are based on pooled data that was collected for the 2009, 2010 and 2012 waves of the German Health Update (GEDA) study by the Robert Koch Institute within the health monitoring framework [24]. The GEDA study is a nationwide telephone survey of German-speaking adults living in private households and having a landline telephone. The data were collected using computer-assisted telephone interviews (CATI). Sampling was carried out in accordance with the Gabler-Häder procedure, whereby a random sample of phone numbers is drawn from all German landline telephone numbers. The sample collated for all three studies amounts to 62,606 people aged 18 or over. The cooperation rate at respondent level, in other words, the number of interviews that were completed after initial contact with a potential participant, was 51.2% in 2009, 55.8% in 2010 and 76.7% in 2012. This shows just how successful the interviewers were in encouraging people to participate in the study. The response rate, in other words, the proportion of completed interviews from the number of neutral non-responses in the adjusted gross sample, amounted to 29.1% in 2009, 28.9% in 2010 and 22.1% in 2012 [24]. The GEDA study was approved by the Federal Commissioner for Data Protection and Freedom of Information. Before an interview began, informed oral consent was obtained.

The analysis presented in this paper was based on a sample restricted to people aged between 18 and 59. Moreover, people were only included in the sample if they lived together with at least one child under the age of 18 in the same household. No distinction was made between biological, step or adoptive children (social parenthood). Plausibility checks were conducted on the ages of people within a household, and this led to the exclusion of 50 respondents. The final analyses, therefore, are based on data provided by 9,806 women and 6,279 men with at least one child under the age of 18 living in the same household. A description of the sample is provided in Table 1.

### 2.2 Variables

The GEDA waves collected data on the health-related variables as follows: data on self-rated general health were gathered by asking the respondents: ‘What is your general state of health like?’ The five response categories were combined into ‘very good/good’ and ‘fair/poor/very poor’. Depression was defined as the presence of medically or psychotherapeutically diagnosed depressive disorders or depressive moods during the last 12 months (12-month prevalence). Data on the 12-month prevalence of back pain was collected by asking the respondents whether they had experienced back pain lasting for at least 3 months during the last 12 months. The data on obesity stem from the self-reported information provided on body size and weight. According to the World Health Organization (WHO), obesity is defined as a body mass index (BMI) of over 30 kg/m^2^. Data on whether a respondent smoked (‘yes’/‘no’) was gathered using the question: ‘Do you currently smoke – even if only occasionally?’ In the analysis presented here, the response categories ‘daily’ and ‘occasionally’ were combined to form the category ‘yes’. The analysis of levels of sporting inactivity is based on the answers provided by respondents who had not practiced sport during the last 3 months (‘yes’/‘no’). Finally, the prevalence of dental check-ups reflects responses to a question about utilization of dental care in the last 12 months (‘yes’/‘no’).

The ‘partner status’ variable was used to differentiate between single parents and parents living with a partner. The respondents were asked whether they lived with a partner in the same household (‘yes’/‘no’) irrespective of marital status or their partner’s sex. The data set contains information on 2,057 single mothers and 235 single fathers with at least one child under the age of 18 living in the same household.

All analyses have been stratified by sex and the following control variables were included in the study: the participants were grouped according to age (18-29 years, 30-39 years, 40-49 years and 50-59 years), according to the number of children in a particular household (‘1 child’, ‘2 children’ and ‘3 or more children’), the age of the youngest child in the household (‘0-6 years’, ‘7-10 years’ and ‘11-17 years’) and residential region (‘old federal states’, and ‘new federal states – including Berlin’). Furthermore, socio-economic status was defined using an index that included information on educational and vocational training, occupational position, and the need-weighted household net income; this enabled respondents to be grouped according to a ‘low’, ‘middle’ or ‘high’ level of socio-economic status [25]. The respondents’ employment status was represented by three categories: ‘full-time employment’, ‘part-time employment’ and ‘non-employed’. Alongside the non-employed, the latter category also includes students, retirees, housewives and househusbands. Finally, the degree of social support that a particular respondent received was measured using the ‘Oslo-3 Social Support Scale’ [26] and categorised as ‘low’, ‘middle’ or ‘high’.

### 2.3 Data analysis

As a first step, prevalences were calculated for all of the selected health indicators for single parents and parents living with a partner (research question 1). The results were stratified according to sex. The odds ratios (also stratified according to sex) were estimated using binary logistic regression. The odds ratio indicates how much more likely it is that a particular health impairment will affect a single parent compared to mothers and fathers living with a partner (the reference group). In this way, it was possible to control for the different composition of the groups of single parents and parents living with a partner (Table 1). By adding stepwise the control variables to the model, it is possible to show which social factors influence the health of single parents (research question 2). Model 1 was adjusted for age, the number of children in the household, the age of the youngest child and residential area. Model 2a also included socio-economic status, model 2b employment status, and model 2c the level of social support. In model 3, all variables were included at the same time.

Finally, a joint model for women and men was estimated that included all of the control variables as well as interaction terms consisting of sex and partner status. An adjusted Wald test was performed for the interaction terms to test whether a different relationship existed between partner status and health among mothers and fathers (research question 3).

The data on single mothers and mothers living with a partner were then stratified according to socio-economic status, employment status and level of social support (research question 4). This was performed by including interactions and estimating predictive margins; these are the predicted probabilities for the health indicators calculated for the individual subgroups in the fully-adjusted model. The results for depression and smoking are presented in graphic form. Due to the small number of cases, no subgroup analysis was performed for single fathers.

All calculations were carried out using a weighting factor that corrects for deviations within the sample from the population structure (as of 31 December 2010) regarding age, sex, education and federal state. P-values lower than 0.05 were considered as statistically significant. Finally, the analyses were calculated using the statistics software Stata SE 14 (StataCorp, College Station, TX, US).

## 3. Results

Table 2 sets out the prevalences for the selected health parameters in comparison of single and partnered parents (research question 1). All health indicators – with the exception of obesity – show significantly higher prevalences for single mothers compared to mothers living with a partner. In the case of single fathers, a significantly higher prevalence was identified for depression, smoking and the non-utilization of dental check-ups. The differences in self-rated general health and back pain associated with partner status among fathers are lower than those identified among mothers and are not statistically significant. A similar pattern was identified for sporting activity among fathers and mothers. However, due to the small number of single fathers in the data, these findings cannot be taken as statistically proven. Finally, no differences were identified in terms of the association between obesity among mothers and fathers and partner status.

Tables 3 and 4 show the extent to which socio-economic status, employment status and social support mediate the relation between single-parent status and health (research question 2). The differences between single and partnered parents described above initially hold true even after controlling for the number and age of children living in the household and residential region (model 1). As shown in Table 3, socio-economic status can statistically explain a certain proportion of the higher health impairment faced by single mothers compared to partnered mothers. As such, once socio-economic status has been included (model 2a), there is no longer a significant link between sporting inactivity and partner status. Neither the inclusion of employment status (model 2b) nor the addition of social support lead to any relevant changes (model 2c). When all control variables are included simultaneously (model 3), however, significant differences continue to exist between single mothers and mothers living with a partner in terms of self-rated general health, depression, back pain, smoking and the non-utilization of dental check-ups. In the case of fathers (Table 4), the inclusion of socio-economic status (model 2a) did not lead to a change in the odds ratios. The odds ratios hardly changed even after the inclusion of employment status (model 2b) and social support (model 2c), meaning that in the fully-adjusted model (model 3) there are still significant results for depression, smoking and the non-utilization of dental check-ups.

In the fully-adjusted models, the only significant result with regard to the interaction of partner status and gender (research question 3) occurs for the utilization of dental check-ups (results not shown). This means that there is a much stronger relationship between single parenthood and a lack of dental check-ups among single fathers than among single mothers. There are no other fundamentally different relationships in terms of partner status and health outcomes between mothers and fathers with regard to any other indicator, even though among fathers some differences in prevalences between single parents and those living with a partner are lower or not statistically significant due to the small number of cases.

**Figure 1:**
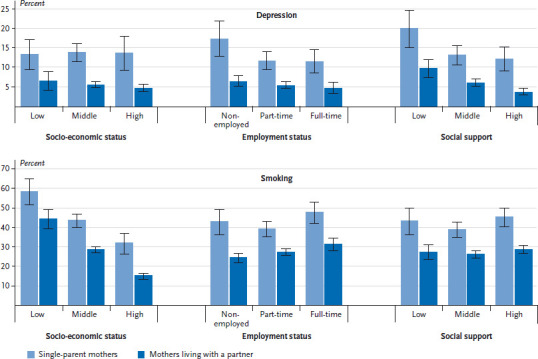
Predicted probabilities of depression and smoking of single mothers and mothers living with a partner, stratified by socio-economic status, employment status and social support (results of binary-logistical regression with interactions) Source: GEDA 2009, 2010, 2012 (pooled)

No significant results were identified for any individual health indicator with respect to the interaction of socio-economic status, employment status, social support and partner status (research question 4) among mothers. This means that partner status in combination with socio-economic status, employment status or social support have a cumulative impact on health. Significant odds ratios for single parenthood as well as for the factors linked to the social situation mentioned above (for the social factors not shown in detail here), are associated with a significantly higher probability of health impairment. Figure 1 shows the predicted probabilities for single mothers and those living with a partner stratified according to socio-economic status, employment status and social support. Each subgroup of single mothers has a higher probability of depression and smoking than mothers living with a partner. In addition, the likelihood of depression is significantly higher among single mothers who are non-employed or who receive low levels of social support than among other subgroups of mothers. A similar picture emerges with regard to smoking when socio-economic status is taken into account: the probability of smoking is highest (just under 60%) among single mothers with a low socio-economic status. At the same time, single mothers with high socio-economic status are less likely to smoke compared to mothers with low socio-economic status who live together with a partner.

## 4. Discussion

Significantly higher prevalences of poor self-rated general health, as well as depression, back pain, smoking, sporting inactivity, and a lack of utilization of dental check-ups were identified among single mothers compared to mothers living with a partner. Higher prevalences of depression, smoking and non-utilization of dental care were identified among single fathers. The prevalence of obesity does not vary among mothers or fathers when examined according to partner status. These results largely coincide with national and international research. A number of studies have shown that single mothers rated their general health poorer than mothers living with a partner [6, 12, 27-35]. However, the differences in the self-assessment of fathers’ general health are somewhat more moderate [27, 28, 36]. Numerous other studies also show a higher prevalence of mental health problems among single mothers [11, 37-42] and single fathers [15, 16, 40, 42]. Several studies report higher prevalences of smoking among single mothers [12, 22, 32, 33, 43-45] and fathers [43]. Results for back pain [6, 29], obesity [8, 33], sporting inactivity [9,12] and the utilization of dental check-ups [46] are relatively rare for single mothers, but those that are available are consistent with the findings described here.

With regard to the question of whether single parenthood affects the health of single mothers and fathers differently, Klose and Jacobi [16] and Wade et al. [42] found no significant differences in terms of the interaction between gender and partner status for mental disorders. Chiu et al. also found no differences between single mothers and fathers with regard to self-rated general and mental health [47]. This is broadly in line with the results presented here: except for the utilization of dental check-ups, the results provide no statistical evidence that supports the theory that the relationship between partner status and health fundamentally differs between mothers and fathers. However, it is important to remember that the statistical power is limited by the relatively small number of single fathers in the data. As the relationship between the utilization of dental check-ups and partner status is much stronger among fathers than mothers, it is possible that partnered men benefit from their female partner’s higher level of health awareness [48].

The results remain largely stable after controlling for the number of children, the age of the youngest child, the residential region, socio-economic status, employment status and social support. The differences in the prevalence between single and partnered parents, therefore, cannot be attributed to differences in social situation. Thus, the health of single parents appears to be largely independent of the social factors taken into account by this study. Nevertheless, different views have been expressed on this issue in the literature. Some studies argue that low levels of financial resources and social support among single parents explain the higher probability of depression [38, 41, 42, 49] and of poor self-rated general health [34, 50]. Other studies, however, show significant health disparities after controlling for socio-economic factors [28, 30, 43, 44, 51] or social support [43, 51]. To a certain extent, the differences between these results seem to be related to the fact that subjectively perceived financial burdens and dissatisfaction with the perceived social support can better explain the differences in the health of single and partnered parents than the comparatively objective data on income or the number of people in a person’s social environment as they were collected in the GEDA study [19]. With regard to employment status, several studies conclude that single mothers have a significantly poorer state of health than mothers living with a partner, irrespective of their employment status [29, 31]. This finding is also consistent with the results presented here.

However, the partially higher health impairment identified among single parents may be less related to single parenthood, per se, and more likely to be linked to previous experiences of conflict during a partnership, as well as a separation or a divorce which may have negative impact on the respondent’s health [52]. Avison et al. have also shown that depression in young adulthood increases the likelihood of separation from a partner [53]. At the same time, the greater psychosocial burden of having sole responsibility for the family and the child/children’s upbringing, as well as conflicts with a former partner can also have an impact on health [54]. Furthermore, a lack of temporal sovereignty can also have an adverse effect on health, especially in the case of single parents in employment [55]. As such, the lower rate of utilization of dental check-ups and the even lower rate of sporting activity could, in fact, be related to a lack of time. However, these aspects are not analysed in the studies mentioned above.

Furthermore, it has been shown that single parents are not a homogenous group; rather their health varies according to socio-economic status, employment status and social support. These social factors are therefore important resources for the health of single parents. Nevertheless, these social resources do not have fundamentally different effect on single parents than on parents living with partners. However, if single parenthood is accompanied by low socio-economic status, non-employment, or a low level of social support, single parents face a cumulative disadvantage in some aspects of their health. The international studies analysing the interaction between single parenthood and employment status among mothers show varying results, probably because of differences in the social systems in which the studies were conducted [31, 32, 35].

Overall, the results of international studies can only be compared to a limited extent due to varying study designs. In addition, they can only be partly applied to the situation in Germany because of the different social systems in which these studies were conducted. International comparisons have produced slightly different results in different western countries [56], and, to a certain extent, this can be attributed to the different social policies [50].

### 4.1 The strengths and weaknesses of this study

One of the strengths of this study is that the size of the sample allows analyses on the health of single mothers and fathers. However, subgroup analyses for single fathers are only possible to a limited extent due to the small number of cases. A further limitation comes from the fact that the GEDA waves are implemented as cross-sectional studies, and thus conclusions cannot be drawn regarding the direction of the association between partner status and health. Additionally, the cross-sectional design makes it impossible to analyse whether single parenthood, or rather a prior separation or divorce, causes health impairment. Similarly, since the GEDA studies do not collect information on the reasons for single parenting, the time of the separation or the duration of single parenthood, no analyses can be conducted on whether and how these factors affect health in the course of life in the short and long term. At the same time, other aspects that could be relevant to the health of single parents were not incorporated into the analysis. This includes a relationship with a partner who does not live in the same household. The study is also unable to account for the diversity among separated parents: it is impossible to identify families where children live in two or more separate households (as part of ‘shared residence’ models). Moreover, it would be wrong to generalise and claim that the health of single parents is, on the whole, worse compared to that of partnered parents. Chronic somatic diseases, for example, which do not occur very widely in young adulthood, were not taken into account as part of this study. Therefore, a more detailed analysis is needed on the living conditions of single parents and further aspects of their health. In the future, differentiated analyses of the social situation and health of single parents are planned and these will account for differences in education, income and occupational position.

### 4.2 Conclusions

Although the vast majority of single mothers and fathers are in good health, single parenting can be associated with health impairments. Negative impacts on health and health-related behaviour of single parents are particularly evident when further disadvantages occur in addition to the existing burden of having sole responsibility for the child/children’s care. The significantly increased risk of poverty among one-parent families is of particular importance and is strongly associated with health impairments. In addition, single parents can also face psychosocial burdens, and these sometimes result from a lower degree of time sovereignty and a lack of social resources.

Single parents are explicitly mentioned as a target group as part of the federal framework recommendations made by the National Prevention Conference in accordance with §20d para. 3 SGB V: ‘Single-parents and their children are particularly often exposed to considerable psychosocial and material burdens due to their living situation and should therefore be given particular consideration in prevention and health promotion measures [57].

A sustainable family policy is essential in promoting the health of single parents. It needs to compensate for the disadvantages that result from the living situation of single parents, and, in particular, reduce the high risk of poverty and levels of psychosocial stress. Bertram et al. [58] argue that sustainable family policy involves transfer payments, as well as time and infrastructure policies.

The overall evaluation of marital and family-related measures and benefits in Germany [59], which was commissioned by the Federal Ministry of Finance and the Federal Ministry of Family Affairs, Senior Citizens, Women and Youth, emphasises benefits such as subsidised childcare, tax relief for single parents, as well as children’s and parental allowance as important aspects of a sustainable family policy. However, the ‘Verband alleinerziehender Mütter und Väter’ notes that the subsidiarity principle stipulated in social law prevents many current family policy-related benefits from contributing sufficiently to reducing poverty among single parents. For example, parental, child and maintenance allowances are all taken into account when calculating the level of social benefits provided to single parents in accordance with SGB II [60]. Therefore, various actors are discussing a move towards a system that provides basic child support [60-64].

In addition to financial transfers, time issues are also important for single parents, as they are particularly affected by the difficulty of reconciling work and family life due to their dual role as breadwinners and primary carers. However, although a higher rate of employment among single parents could reduce the risk of poverty, it would also leave them with less time for their family. Therefore, further flexibilisation of working time models over the course of a person’s life, which could also include a phase of reduced working hours or even temporary leave from work, that take into account the different time requirements in different phases of life would be more useful [61]. Moreover, a good level of child care infrastructure is particularly important as it enables single parents to reconcile work and family life. The ‘Verband alleinerziehender Mütter und Väter’ argues that improving the quantity and quality of child care, expanding all-day schools and providing out-of-hours childcare are particularly important [65].

Family policy incentives could encourage parents to divide equally the responsibilities for the family, even in the case of separated or divorced couples, and, as such, promote a more responsible form of shared parenthood. In addition to family models in which a child predominantly remains with one parent, other models are increasingly developing in which a child lives with each parent at different times, leading to a fairer division of care between the parents. However, this model can be impractical, especially in cases of severe conflicts between separated parents. Moreover, legal frameworks and family policies have yet to be adequately tailored to the needs of these families [66].

According to Geene and Töpritz, integrated activities provided by local authorities in the context of ‘prevention chains’ can help to build multi-professional and inter-sectoral networks and thus make available more transparent, tailor-made and accessible offers for socially disadvantaged parents [67]. Local services, such as the Early Intervention Programme called „Frühe Hilfen“, family centres, counselling and mediation, as well as measures provided by youth welfare or employment agencies, can reach single and partnered parents with socially induced unequal health-related opportunities without subjecting them to stigmatisation in their everyday life situations [57, 67].

In summary, low-threshold and setting-based measures combined with better financial protection for single-parent families, and improved opportunities to reconcile work and family life, could play an important role in promoting the health of single parents.

## Key statements

- The proportion of single-parent families compared to the total number of families in Germany has increased significantly in the past few decades and amounted to 20.3% in 2014.
- Single parenthood is associated with substantial demands on parents and can also result in health impairment.
- Single parents report more frequently that they have been medically diagnosed with depression and smoke more often than mothers and fathers living with a partner.
- Low socio-economic status, a lack of paid employment and a low level of social support can increase the health impairment of single parents.
- Improving the financial position of single-parent families and making it easier to reconcile work and family life are important steps that would help improve the health of single parents.

## Figures and Tables

**Table 1:** Description of the sample: single parents and parents living in partnership according to age, socio-economic status, employment status and social support (frequencies in %, 95% confidence intervals) Source: GEDA 2009, 2010, 2012 (pooled)

	Mothers				Fathers			
	Single parents		Living in partnership		Single parents		Living in partnership	
n (unweighted)	2,057		7,749		235		6,044	
*%* (weighted)	14.0%		86.0%		2.3%		97.7%	
**%**		**(95% CI)**	**%**	**(95% CI)**	**%**	**(95% CI)**	**%**	**(95% CI)**
**Age**								
18-29 Years	13.1	(11.1-15.4)	10.5	(9.6-11.4)	7.4	(3.7-14.2)	5.1	(4.4-5.9)
30-39 Years	29.9	(27.6-32.3)	42.7	(41.4-44.0)	24.4	(17.5-32.9)	32.8	(31.3-34.3)
40-49 Years	46.8	(44.2-49.5)	40.8	(39.6-42.1)	51.9	(43.7-60.0)	48.3	(46.7-49.8)
50-59 Years	10.2	(8.7-12.0)	6.0	(5.4-6.7)	16.3	(11.4-22.8)	13.9	(12.8-15.0)
**Socio-economic status**								
Low	29.5	(26.8-32.4)	14.6	(13.5-15.9)	20.4	(13.9-28.7)	18.9	(17.4-20.5)
Middle	59.6	(56.8-62.3)	60.6	(59.3-61.9)	61.1	(52.9-68.8)	55.2	(53.6-56.7)
High	10.9	(9.7-12.2)	24.7	(23.8-25.7)	18.5	(13.7-24.6)	25.9	(24.8-27.1)
**Employment status**								
Non-employed	24.1	(21.7-26.7)	29.8	(28.6-31.1)	11.6	(7.2-18.3)	6.4	(5.6-7.4)
Part-time employment	46.3	(43.6-48.9)	53.3	(52.0-54.6)	11.0	(7.0-16.9)	5.5	(4.9-6.3)
Full-time employment	29.6	(27.3-32.1)	16.9	(15.9-17.9)	77.4	(69.8-83.5)	88.0	(86.9-89.1)
**Social support**								
Low	21.9	(19.6-24.4)	13.4	(12.4-14.4)	15.9	(11.0-22.4)	12.9	(11.7-14.1)
Middle	46.6	(43.9-49.3)	49.6	(48.3-50.9)	52.0	(43.6-60.2)	49.8	(48.2-51.3)
High	31.5	(29.1-34.1)	37.1	(35.8-38.3)	32.2	(24.8-40.5)	37.4	(35.9-38.9)

CI=confidence interval

**Table 2:** Health and health-related behaviour of single parents and parents living in partnership (prevalence, 95% confidence interval) Source: GEDA 2009, 2010, 2012 (pooled)

			Mothers		Fathers	
	n	Single parent	%	(95% CI)	%	(95% CI)
Self-rated health (fair – very poor)	16,075	Yes	**25.5**	**(23.1-28.0)**	23.4	(16.8-31.7)
		No	**17.0**	**(16.0-18.1)**	17.5	(16.2-18.8)
Depression	16,051	Yes	**15.0**	**(13.2-16.9)**	**12.4**	**(7.6-19.6)**
		No	**6.0**	**(5.4-6.7)**	**4.6**	**(3.9-5.4)**
Back pain	16,071	Yes	**24.0**	**(21.7-26.5)**	17.9	(12.1-25.5)
		No	**17.7**	**(16.7-18.8)**	14.5	(13.4-15.7)
Obesity	15,813	Yes	12.6	(10.8-14.6)	16.5	(10.7-24.5)
		No	11.9	(10.9-12.9)	14.6	(13.5-15.8)
Smoking	16,083	Yes	**48.6**	**(45.9-51.3)**	**50.4**	**(42.3-58.5)**
		No	**27.6**	**(26.4-28.8)**	**36.8**	**(35.3-38.4)**
Sporting inactivity	16,076	Yes	**37.1**	**(34.4-39.8)**	37.9	(30.2-46.3)
		No	**32.8**	**(31.5-34.1)**	32.1	(30.6-33.6)
Non-utilization of dental care	16,065	Yes	**19.8**	**(17.5-22.2)**	**40.3**	**(32.3-48.9)**
		No	**14.3**	**(13.3-15.4)**	**24.9**	**(23.5-26.3)**

In bold: significant (p<0.05); CI=confidence interval

**Table 3:** Health and health-related behaviour of single mothers compared to mothers living in partnerships (results of binary logistic regression, odds ratios, 95% confidence intervals) Source: GEDA 2009, 2010, 2012 (pooled)

Mothers	Model 1		Model 2a (+ SES)		Model 2b (+ employment status)		Model 2c (+ social support)		Model 3 (fully adjusted)	
	OR	(95% CI)	OR	(95% CI)	OR	(95% CI)	OR	(95% CI)	OR	(95% CI)
Self-rated health (fair – very poor) (n=9,569)	**1.57**	**(1.34-1.83)**	**1.32**	**(1.12-1.54)**	**1.58**	**(1.35-1.85)**	**1.44**	**(1.23-1.69)**	**1.26**	**(1.07-1.49)**
Depression (n=9,549)	**2.77**	**(2.28-3.36)**	**2.58**	**(2.10-3.18)**	**2.82**	**(2.32-3.43)**	**2.59**	**(2.12-3.15)**	**2.55**	**(2.06-3.15)**
Back pain (n=9,565)	**1.44**	**(1.23-1.67)**	**1.27**	**(1.09-1.49)**	**1.44**	**(1.23-1.67)**	**1.36**	**(1.17-1.59)**	**1.23**	**(1.05-1.45)**
Obesity (n=9,342)	1.10	(0.90-1.35)	0.87	(0.70-1.08)	1.10	(0.90-1.35)	1.06	(0.86-1.30)	0.85	(0.69-1.05)
Smoking (n=9,570)	**2.30**	**(2.02-2.62)**	**1.97**	**(1.72-2.25)**	**2.27**	**(1.99-2.59)**	**2.27**	**(1.99-2.59)**	**1.92**	**(1.67-2.20)**
Sporting inactivity (n=9,567)	**1.32**	**(1.15-1.51)**	1.07	(0.93-1.24)	**1.30**	**(1.14-1.50)**	**1.25**	**(1.09-1.44)**	1.04	(0.90-1.20)
Non-utilization of dental care (n=9,557)	**1.57**	**(1.31-1.88)**	**1.32**	**(1.09-1.59)**	**1.56**	**(1.30-1.87)**	**1.49**	**(1.24-1.78)**	**1.28**	**(1.06-1.54)**

In bold: significant (p<0.05) All models adjusted for age, number of children, age of the youngest child and residential area. Reference group: mothers living with a partner; CI=confidence interval, SES=socio-economic status

**Table 4:** Health and health-related behaviour of single fathers compared to fathers living with a partner (results of binary logistical regressions, odds ratios, 95% confidence intervals) Source: GEDA 2009, 2010, 2012 (pooled)

Fathers	Model 1		Model 2a (+ SES)		Model 2b (+ employment status)		Model 2c (+ social support)		Model 3 (fully adjusted)	
	OR	(95% CI)	OR	(95% CI)	OR	(95% CI)	OR	(95% CI)	OR	(95% CI)
Self-rated health (fair – very poor) (n=6,117)	1.33	(0.86-2.06)	1.29	(0.82-2.04)	1.21	(0.74-1.98)	1.28	(0.82-2.00)	1.16	(0.69-1.94)
Depression (n=6,115)	**2.65**	**(1.44-4.89)**	**2.62**	**(1.43-4.80)**	**2.36**	**(1.27-4.38)**	**2.54**	**(1.37-4.69)**	**2.23**	**(1.20-4.16)**
Back pain (n=6,116)	1.26	(0.78-2.04)	1.23	(0.76-2.00)	1.20	(0.73-1.97)	1.22	(0.75-2.01)	1.15	(0.69-1.92)
Obesity (n=6,100)	1.14	(0.67-1.93)	1.11	(0.64-1.91)	1.14	(0.67-1.95)	1.12	(0.65-1.92)	1.12	(0.64-1.94)
Smoking (n=6,122)	**1.67**	**(1.17-2.37)**	**1.63**	**(1.14-2.31)**	**1.60**	**(1.13-2.27)**	**1.66**	**(1.17-2.37)**	**1.59**	**(1.12-2.26)**
Sporting inactivity (n=6,120)	1.29	(0.90-1.85)	1.24	(0.87-1.78)	1.25	(0.88-1.79)	1.26	(0.88-1.82)	1.21	(0.84-1.74)
Non-utilization of dental care (n=6,119)	**2.29**	**(1.58-3.31)**	**2.27**	**(1.54-3.34)**	**2.28**	**(1.57-3.30)**	**2.25**	**(1.55-3.27)**	**2.27**	**(1.54-3.34)**

In bold: significant (p<0.05) All models adjusted for age, number of children, age of the youngest child and residential area. Reference group: fathers living with a partner; CI=confidence interval, SES=socio-economic status
